# Efficacy of Radioiodine Therapy in Patients With Primary Hyperthyroidism: An Institutional Review From Pakistan

**DOI:** 10.7759/cureus.24992

**Published:** 2022-05-14

**Authors:** Asim Munir Alvi, Umal Azmat, Waqas Shafiq, Abdul Hannan Ali Rasheed, Ahmed Imran Siddiqi, Sardar Khan, Sara Ashfaq, Hira Irfan, Humayun Bashir, Muhammad Abu Bakar, Kashif Asghar

**Affiliations:** 1 Endocrinology, Diabetes and Metabolism, Shaukat Khanum Memorial Cancer Hospital and Research Centre, Lahore, PAK; 2 Nephrology and Hypertension, Loyola University Medical Center, Chicago, USA; 3 Nuclear Medicine, Kent and Canterbury Hospital, Canterbury, GBR; 4 Biostatistics and Epidemiology, Shaukat Khanum Memorial Cancer Hospital and Research Centre, Lahore, PAK; 5 Basic Sciences, Shaukat Khanum Memorial Cancer Hospital and Research Centre, Lahore, PAK

**Keywords:** toxic multinodular goiter, toxic adenoma, graves’s diseases, radioiodine, primary hyperthyroidism

## Abstract

Background

Radioactive iodine (RAI) is the treatment of choice for most patients with primary hyperthyroidism. The most common etiologies of hyperthyroidism are Graves' disease (GD), toxic adenoma (TA), and toxic multinodular goiter (TMNG). A single dose of RAI is usually sufficient to cure hyperthyroidism. The aim of this study was to assess the effectiveness of RAI therapy for patients diagnosed with primary hyperthyroidism.

Methods and materials

Patients diagnosed with hyperthyroidism who received RAI therapy between 2008 and 2018 were included in the study. The data was acquired from the hospital's electronic medical record system. Following the RAI treatment, a cure was defined as the development of euthyroidism or hypothyroidism after a single fixed-dose without antithyroid medication within one year of RAI therapy. In addition, a simple logistics regression model was used to identify the prognostic factors that may lead to better outcomes.

Results

A total of 112 patients diagnosed with hyperthyroidism with a mean age of 47 ± 14 were included in this study. The majority of the patients were female, 79 (70.5%). Within one year of RAI therapy, 84 (75%) patients achieved a cure that is either hypothyroid or euthyroid status. RAI dose was higher in responsive patients (18.50 ± 4.10 millicurie [mCi] versus 16.50 ± 4.10 mCi) than in non-responsive patients. The mean RAI doses were 16.05 ± 2.99 mCi in GD, 19.81 ± 4.40 mCi in TMNG, and 20.50 ± 3.30 mCi in TA, with a statistically significant p-value of 0.001. In the univariable logistic regression model, RAI dose was a significant prognostic factor of the responsive group (OR: 1.15, CI [1.01-1.31], p-value 0.03).

Conclusion

Our data presented that RAI therapy is effective for primary hyperthyroidism. We achieved remission with a single fixed-dose in the majority of patients. Most of our patients were cured within three months of RAI therapy. In addition, the RAI dose was higher in the responsive group as compared to the non-responsive group.

## Introduction

Hyperthyroidism is an endocrine disorder [1] caused by the overproduction of thyroid hormone [2]. Hyperthyroidism is a biochemical diagnosis, and thyrotoxicosis is a clinical diagnosis related to its symptoms [3]. The most common etiology of hyperthyroidism is Grave's disease (GD), followed by toxic multinodular goiter (TMNG) and toxic adenoma (TA) [2, 3, 4]. Hyperthyroidism can be managed with antithyroid drugs (ATDs), radioactive iodine (RAI), or surgery [2, 3]. Each therapeutic modality has its own advantages and disadvantages. ATDs are commonly used as the first-line treatment in Europe and as RAI therapy in North America [2, 5-7]. A recent study has reported that ATDs have a higher failure rate of about 52.7% in GD [8]. The significant but rare side effects of ATDs are agranulocytosis [9, 10], hepatotoxicity, pancreatitis, and vasculitis [4, 11, 12]. Around 15% of people may experience minor side effects: itching, rash, hives, joint pain, swelling, fever, changes in taste, nausea, and vomiting [2, 4, 12]. The overall cost of treatment and risk of recurrence of hyperthyroidism is higher with ATDs than with RAI and surgery [13]. Benefits include fewer chances of development of hypothyroidism or aggravation of thyroid eye disease [11].

In most patients with hyperthyroidism, the definitive treatment is RAI or surgery [4, 14]. Surgery is preferred in patients with compressive symptoms [2], poor compliance to medical treatment, pregnant females who are not responsive to the ATDs, and patients with severe Graves ophthalmopathy [3, 4]. Compared to the RAI therapy, the surgery has a risk of more complications, including wound infection, hemorrhage, laryngeal edema, hypoparathyroidism, recurrent laryngeal nerve palsy, and thyroid storm [15, 16].

It has been reported that RAI therapy is generally safe and well-tolerated [2, 17]. RAI therapy for hyperthyroidism is usually recommended for individuals who are intolerant or resistant to ATDs, those with an increased risk of surgery, and those with previously operated or externally irradiated necks [3, 18]. Previously published data demonstrated that the efficacy of RAI therapy with a single dose is around 66%-93% [19-21]. Disease severity at the time of presentation, younger age, male gender, and use of ATDs are the factors that may affect the outcomes of RAI single-dose therapy [19, 22, 23].

Six to eighteen weeks are usually required to get favorable outcomes (either euthyroid or hypothyroid) after RAI therapy [23]. There are two common practices regarding the dose of RAI therapy, fixed-dose and dosimetric [4, 24]. There is a discrepancy in the methodology of the RAI therapy. Our study aim was to assess the effectiveness of RAI therapy for patients diagnosed with primary hyperthyroidism and treated at Shaukat Khanum Memorial Cancer Hospital and Research Center (SKMCH&RC) in Lahore, Pakistan.

## Materials and methods

The institutional review board (IRB; study IRB# EX-22-01-19-02) of Shaukat Khanum Memorial Cancer Hospital and Research Center (SKMCH&RC) has approved the current retrospective study. IRB-SKMCH&RC allowed the waiver for informed consent for this study. We reviewed the medical records of all patients with hyperthyroidism who received RAI therapy from June 2008 to May 2018. Patient demographics, diagnosis, complications of hyperthyroidism, use of antithyroid medications before and after RAI therapy, the dose of RAI, and treatment efficacy were collected from the electronic medical record system called the hospital information system (HIS).

The etiology of hyperthyroidism was based on clinical findings, thyroid function tests, and thyroid scans. Patients were given the diagnosis of GD when they had thyroid-associated ophthalmopathy, suppressed TSH with elevated free T4, free T3, or diffuse symmetrical increased uptake on a 99m technetium isotope scan. Patients with TMNG had a multinodular goiter on examination (those without palpable goiter clinically were also included) and suppressed TSH with high free T4, free T3, and variable uptake in nodules on a 99m technetium scan. Patients with TA were diagnosed with palpable nodule (those without palpable nodule clinically were also included) and suppressed TSH with high free T4 and free T3 and hot nodule with suppressed background activity on a 99m technetium scan.

We used the RAI fixed dose method in our patients. Patients on antithyroid medications were asked to hold them for 5-7 days prior and to recommence five days following RAI therapy. A nuclear medicine physician estimated the RAI dose based on the following parameters: (1) type of hyperthyroidism; and (2) percentage uptake on pre-therapy uptake scan. Patients were followed with thyroid function tests every 4-6 weeks initially and then 2-3 monthly for 12 months to assess the outcome of RAI therapy.

Therapy was considered successful if remission, defined as the development of hypothyroidism (high TSH with low free T4) requiring treatment with levothyroxine or euthyroidism (normal TSH with normal free T4) without ATDs was achieved within 12 months of RAI therapy. Conversely, if the patient remained hyperthyroid (low TSH, high free T4) after RAI therapy and/or required either a repeat session of RAI or long-term ATDs to achieve euthyroidism, they were considered as a treatment failure.

Statistical analysis

Statistical analysis was carried out using the SPSS software (version 20.0; SPSS, Chicago, IL, USA). Continuous variables were stated as mean ± SD, and categorical variables were computed as frequencies and percentages. Categorical variables were compared using the Chi-squared test or Fisher's exact test (when necessary). The continuous variables were compared using the independent t-test. One-way ANOVA was used to check the mean difference among three different diagnoses. Statistical significance was defined as a two-tailed p-value of 0.05.

## Results

Demographic and clinical characteristics

A total of 114 patients were reviewed, and 112 subjects met the criteria for the study. Two subjects were excluded because of less than 18 years of age. Among the 112 subjects, 79 (70.5%) were females, and 33 (29.5%) were males. The mean age of the sample was 47 ± 14 years, and the mean weight was 68 ± 17 Kg. On subgroup analysis, the mean age of the patients with GD was 43 ± 13 years, with TMNG was 53 ± 15 years, and with TA was 46 ± 11 years. The mean weight of the patients with GD was 68 ± 18 kg, with TMNG was 68 ± 15 kg, and TA was 66 ± 14 kg. Based on clinical, laboratory, and thyroid scan results, 57 (50.9%) patients had a diagnosis of GD, while TMNG and TA were diagnosed in 36 (32.1%) and 19 (17.0%) patients, respectively. While 100 (89.3%) patients belonged to the Punjab province, 7 (6.3%) were from Khyber Pakhtunkhwa (KPK), 2 (1.8%) from Balochistan, 2 (1.8%) from Afghanistan, and 1 (0.9%) from Islamabad. Most of them (89.2%) were already taking ATDs when they were referred to our institute for RAI therapy. At the time of presentation, 106 (94.6%) patients were hyperthyroid, and 6 (5.4%) were euthyroid (Table 1).

**Table 1:** Baseline characteristics of patients diagnosed with primary hyperthyroidism. mCi: millicurie; RAI: Radioactive iodine; KPK: Khyber Pakhtunkhwa.

Variables	Categories	N (%)
Age	Mean ± SD	47 ± 14
Weight at presentation	Mean ± SD	68 ± 17
Dose in mCi of I^st^ RAI	Mean ± SD	18 ± 4
Gender	Male	33 (29.5)
	Female	79 (70.5)
Province	Afghanistan	2 (1.8)
	Balochistan	2 (1.8)
	Islamabad	1 (0.9)
	KPK	7 (6.3)
	Punjab	100 (89.3)
Diagnosis	Grave’s disease	57 (50.9)
	Toxic multinodular goiter	36 (32.1)
	Toxic adenoma	19 (17.0)
Outcome at presentation	Euthyroid	6 (5.4)
	Hyperthyroid	106 (94.6)
Outcome achieved after 1st RAI	Not achieved	28 (25.0)
	Achieved	84 (75.0)

Outcomes of RAI therapy

The outcome of RAI therapy was achieved in 84 (75%) patients after the first dose (Table 1). The mean dose of the RAI therapy was 18±4 mCi. There is a statistically significant (p-value=0.03) mean difference in RAI dose in patients who achieved an outcome (18±4 mCi) and those who did not achieve an outcome (17±4 mCi). Furthermore, our data showed that the majority, 68 (80.9%), achieved outcomes (euthyroid or hypothyroid) within three months of RAI therapy (Table 2).

**Table 2:** Demographic and clinical characteristics of primary hyperthyroidism with respect to outcome. mCi: millicurie; RAI: Radioactive iodine; KPK: Khyber Pakhtunkhwa.

Variables	Categories	Outcome after RAI		P-value
		Not achieved 28 (25.0%)	Achieved 84 (75.0%)	
Age	Mean ± SD	48 ± 14	46 ± 14	0.7
Weight at presentation	Mean ± SD	72 ± 22	67 ± 14	0.16
Dose in mCi of 1st RAI	Mean ± SD	17 ± 4	18 ± 4	0.03
Duration (months)	Mean ± SD	6 ± 4	11 12	0.04
Gender	Male	9 (32.1)	24 (28.6)	0.72
	Female	19 (67.9)	60 (71.4)	
Province	Afghanistan	0 (0.0)	2 (2.4)	
	Balochistan	1 (3.6)	1 (1.2)	
	Islamabad	0 (0.0)	1 (1.2)	
	KPK	3 (10.7)	4 (4.8)	
	Punjab	24 (85.7)	76 (90.5)	
Diagnosis	Grave’s disease	12 (42.9)	45 (53.6)	0.16
	Toxic multinodular goiter	13 (46.4)	23 (27.4)	
	Toxic adenoma	3 (10.3)	16 (19.0)	
Outcome at presentation	Euthyroid	1 (3.6)	5 (6.0)	1
	Hyperthyroid	27 (96.4)	79 (94.0)	
Time to achieve outcome	Not achieved	28 (100.0)	84 (100.0)	
	Within 6 weeks	-	39 (46.4)	
	Within 3 months	-	29 (34.5)	
	Within 6 months	-	10 (11.9)	
	Within 9 months	-	3 (3.6)	
	Within 12 months	-	3 (3.6)	

Hypothyroidism developed in 43 (51.1%), and 38 (88.3%) became hypothyroid within three months. Additionally, we observed the mean difference in age, weight (kg), and dose of first RAI among three different diagnoses, i.e., GD, TMNG, and TA. The dose of RAI (p-value = 0.001) and age (p-value = 0.005) showed a statistically significant difference (Table 3).

**Table 3:** Demographic and clinical characteristics of patients diagnosed with GD, TMNG, and TA. mCi: millicurie; RAI: Radioactive iodine; GD: Grave's disease; TMNG: Toxic multinodular goiter; TA: Toxic adenoma.

Variables	Diagnosis			P-value
	GD: 57 (50.9%)	TMNG: 36 (32.1%)	TA: 19 (17.0%)	
Age				0.005
Mean ± SD	43 ± 13	53 ± 15	46 ± 11	
Weight at presentation (kg)				0.89
Mean ± SD	68 ± 18	68 ± 15	66 ± 14	
Dose in mCi of 1st RAI				0.001
Mean ± SD	16.05 ± 3.00	19.81 ± 4.39	20.47 ± 3.30	

A total of 57 (50.9%) cases were diagnosed with GD, and out of these, 45 (79%) cases achieved outcomes subdivided as hypothyroid 32 (71.1%) and euthyroid 13 (28.9%). Additionally, 36 (32.1%) and 19 (17%) cases were diagnosed with TMNG and TA. Therefore, in TMNG and TA groups, 23 (63.9%) and 16 (84.2%) cases showed positive outcomes, respectively. Furthermore, in the TMNG group, 9 (39.1%) and 14 (60.9%) exclusively accomplished as hypothyroid and euthyroid, respectively. Besides that, in the TA group, the outcome of hypothyroid and euthyroid was obtained in 2 (12.5%) and 14 (87.5%) patients, respectively (Table 4).

**Table 4:** Outcomes achieved as hypothyroid or euthyroid in GD, TMNG, and TA. GD: Grave's disease; TMNG: Toxic multinodular goiter; TA: Toxic adenoma.

Variables	Diagnosis		
	GD: 57 (50.9%)	TMNG: 36 (32.1%)	TA: 19 (17.0%)
Not achieved	12 (21.1)	13 (36.1)	3 (15.8)
Hypothyroid	32 (56.1)	9 (25.0)	2 (10.5)
Euthyroid	13 (22.8)	14 (38.9)	14 (73.7)

## Discussion

RAI therapy has been used to treat primary hyperthyroidism for more than 70-80 years with the intent to cure thyrotoxicosis [19, 21, 25-26]. The outcome of RAI therapy varies between 50 and 90% after one year of treatment [19, 27]. Our data showed that 75% of patients were cured with a single dose of RAI, which agrees with already published data globally in different cohorts [1, 17, 19, 20, 23, 28]. Previously published data has contradictory results regarding the efficacy of RAI therapy. It may be due to the differences in the study designs, patient selection, sample size, variability in dose calculation, and ethnicity. The way to respond to the RAI therapy may vary among individuals, which could be the major factor in predicting the outcome. The effectiveness of RAI depends on the iodine retaining and concentrating ability of the thyroid tissue [27]. The research data of some studies showed that the use of lithium before RAI increase the effectiveness of therapy because lithium, without interfering with RAI uptake, can block the RAI release from the thyroid gland [29-32]. The use of lithium with RAI also causes rapid control of hyperthyroidism [27, 33, 34].

Additionally, the study published by Bogazzi F et al. reported that the use of lithium with RAI therapy increased the efficacy and the cure rate by 11%. Furthermore, they observed the early cure of hyperthyroidism [34]. However, Bal CS et al. reported that RAI therapy in combination with lithium has no significant benefit [35]. The RAI therapy given at our hospital was without the use of lithium. Further studies are warranted to identify the role of lithium as an adjuvant therapy with RAI to improve outcomes.

Thyroid disorders are more prevalent in females [2, 36]. In our data, the majority of patients were females (70.5%). We observed no statistical significance of gender on treatment outcome, which is consistent with other studies [1, 37, 38]. Furthermore, the effect of age on the outcome of RAI therapy is contradictory as well. Many previously published studies demonstrated that age did not affect the outcome of RAI [1, 37-38]. Our results were consistent with these previous studies, and we observed that age has no statistical significance on the outcome. In the current study, we observed that a higher dose of RAI was associated with better outcomes. Aldahmani KM et al. [17] reported similar results. Previous data revealed that a higher RAI dose was associated with more outcomes and decreased time to achieve cure [39]. The incidence of hypothyroidism was high in patients receiving a higher dose of RAI than those who received a lower dose [40]. Most of our patients achieved desirable outcomes in six weeks to three months following RAI therapy. Our finding is in accordance with other studies [17, 23]. This finding proposes the importance of early follow-up at 4-6 weeks intervals initially post RAI therapy. This is done to access patients both biochemically with thyroid function tests (TSH, free T3, and Free T4) and clinically for 3 to 6 months or until the patient becomes hypothyroid and is stable on thyroid hormone replacement. After this, patients can be followed up at 3-6 monthly intervals.

Our current data demonstrated that the patients with GD achieved outcomes of hypothyroidism more frequently than TA and TMNG. In contrast, patients with TMNG and TA were more likely to develop outcomes of euthyroidism. Our results are consistent with the previously published data [2, 17, 21]. This difference in response to RAI therapy is ascribed to the sparing effect of RAI therapy on the suppressed nontoxic normal thyroid tissue in TMNG and TA [17]. Additionally, we do not find any severe complications of RAI therapy like thyroid storm and any new development of thyroid ophthalmopathy.

There are a few limitations of our study. First, we could not evaluate the serum levels of TSH receptor antibodies (TRAb). Secondly, we also could not examine the association of the duration of ATDs used before RAI with the outcome. One more limitation of the study was the inherent study design. Retrospective studies are considered low-quality in the hierarchy of evidence due to lack of blindness. Therefore, they have a high potential for recall, reconfirmation, and selection bias. Overall, this may result in false-positive associations.

Nonetheless, all of the information was extracted from the computerized hospital database, and data from other medical providers were correlated to reduce the risk of recall and reporting bias. Another limitation is that this was a single-center study, which may affect the generalizability of the findings. However, this study was conducted in a tertiary care center, where patients were referred for treatment from across the region, including Afghanistan. Nevertheless, these results need to be interpreted with caution and necessitate replication. Future investigations with prospective study design, multi-center enrollment, and larger sample sizes, which use adjuvant treatments in high-risk groups, are advocated.

## Conclusions

In conclusion, our data presented that RAI therapy is effective for primary hyperthyroidism. We achieved remission with a single fixed-dose in the majority of the patients. Most of our patients were cured (achieved the outcome of euthyroidism or hypothyroidism) within three months of RAI therapy. RAI dose was also higher in the responsive group than in the non-responsive group. Therefore, RAI therapy with the appropriate dosage may have a significant outcome in treating primary hyperthyroidism.
